# COVID-19 and the Elderlies: How Safe Are Hong Kong's Care Homes?

**DOI:** 10.3389/fpubh.2022.883472

**Published:** 2022-04-04

**Authors:** Mohana Das

**Affiliations:** ^1^The Hong Kong Polytechnic University, Kowloon, Hong Kong SAR, China; ^2^Politecnico di Milano, Milan, Lombardy, Italy

**Keywords:** COVID-19, aging population, older people, care homes for older people, dynamic zero-COVID, indoor environment, planning and design, policy and guidelines

## Introduction

Even after innumerous advancements in technology and an increase in the average life-expectancy of the humans, the world remained underprepared to deal with the pandemic. This is particularly true in light of the continuing COVID-19 pandemic, which challenged the healthcare system with one of its most critical opportunities to examine its preparedness to deal with the crushing strain of such emergencies around the world in current history. And, as was to be anticipated, the majority of the countries failed their citizens, emphasizing the need to update and re-evaluate the existing policies and infrastructure in the healthcare industry.

The older adults and children were disproportionately affected by the pandemic and were classed as a high-risk group for the disease (1), based on evidence that it targeted the population unequally. This highlighted the significance of having stronger guidelines in place for the vulnerable, who require extra attention, particularly during times of crisis. The ongoing pandemic highlighted in numbers that a high proportions of the death due to COVID were registered amongst the older adults in the USA, Canada, and in European Union states (2, 3).

## Case of Hong Kong: City—COVID—It's Elderlies

### City

Hong Kong, a special administrative region of China, is bordered by mainland China. It has a population of over 7.58 million and the seventh busiest airport in the world, with over 70 million passengers per year. Hong Kong has also one of the world's highest population densities (6887.95/km^2^) (4). All of these factors make Hong Kong particularly vulnerable to the coronavirus disease 2019 (COVID-19) pandemic (5). Yet, unlike the rest of the economies that have been contending with the pandemic since early 2020, Hong Kong has been able to keep itself safe for a long time owing to the strict and timely border restrictions, quarantine measures and a loyal citizenry that has dealt with SARS before.

### COVID

The authorities' “dynamic-zero” approach, which appeared to be working for over 2 years, is now facing its toughest test with an unprecedented surge in the number of infected cases as the fifth wave hits, which began in early 2022 and is rapidly spreading after the Chinese Lunar New Year celebrations- a traditional time primarily for family gatherings (6). For the region, the “living with COVID” strategy that has been adopted by the majority of countries was never an option. Instead, the responsible authorities chose to adhere to the ambitious “net-zero COVID” policy, also adopted by mainland China, which focuses on maintaining zero cases or taking absolute steps to eliminate the possibility of any infected cases in order to keep its people safe and maintain normal activities. This strategy of elimination worked successfully for nearly 2 years following the virus's emergence in Hong Kong (January, 2020), but recently the number of infected cases has skyrocketed, indicating a different dire situation (6). While it took over 2 years and four waves to surpass the 12,000 confirmed cases mark, it has multiplied more than thirty-five times in <2 months in the most recent fifth wave, which is on track to surpass 500,000 confirmed cases and beyond, with 440,609 instances reported on 05 March 2022, with over 200 related death per day being registered (7).

### Elderlies

The biggest fear within the loop of actions taken so far is, first, the lowest inoculation rate in the older population of Hong Kong (7), which is ranked amongst the lowest in the list of developed cities index and secondly, the condition of the highly dense packed vertical lifestyle. The intersection of these two critical components has the potential to create the worst-case catastrophe in the coming days. The unique situation, specially owing to its urban typology will make it difficult to either stress on any one of the strategies of “zero COVID” or “living with COVID,” rather a very local context specific solution needs to be figured out and very soon at that to mitigate the soaring numbers of the infected cases (8, 9). As the world gets older, demand for services that cater to this segment of the baby boomer population is increasing, and this trend will continue in the future years. Among these are elderly care homes, day care centers, and other institutions such as nursing homes, which provide residential care, meals, personal care, regular basic medical and nursing care, and social support to the majority of the older people in Hong Kong and throughout the world (10).

## The Quandary

**Figure 1:**
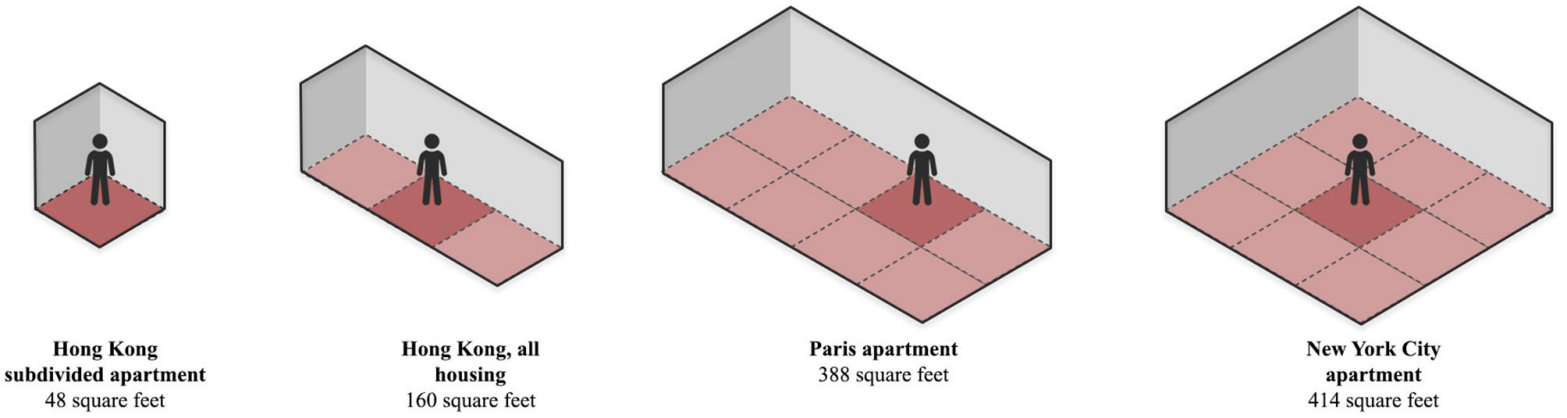
Average living space per person around different cities.

Hong Kong's average living space per capita is approximately 160 square feet. It is 130 square feet in public rental homes—still significantly larger than the 48 square feet in subdivided flats. It is, however, minuscule in comparison to other countries or cities (Refer Figure 1) (11). Adding to this, as the silver population is on the rise with one of the highest life expectancies in the world, the Census and Statistics Department expects over 30% of the population to be above 65 by 2041 (4). At the moment, when the majority of elders with suitable financial means prefer to live in care homes, the epidemic entails the greatest paradox of confined places and increased transmission potential.

Given this scenario, the majority of families who live in a cramped micro apartment may face the most difficulty in self-isolating without the risk of future viral transmission within the family. The current spike has strained the healthcare system, and the following policy revisions propose that moderate or asymptomatic cases need not be admitted to the quarantine centers or hospitals in order to save beds for severe cases (12). In such a case, the elderly care homes in Hong Kong, which often have open floor plans and most of their bed arrangements on a single floor, constitute a significant concern due to the lack of any spatial considerations that would prevent cross-contamination. Numerous studies to date demonstrate that the Omicron variant is significantly more contagious than the preceding ones, despite the reduced fatality rates (13). This might indicate that the virus, which is already more dangerous for seniors due to its high (aerial) transmissibility, along with unfavorable capacity planning and low inoculation rates, could spark a breakout within the care homes and further add to the crisis.

## The COVID-19 Policy Road Map

As observed in the past four waves, the strict adherence to the “net-zero COVID” policy resulted in one of the lowest infected and death rates in the world for almost 2 straight years while the rest of the world faced the severity of the pandemic. The policy was widely applauded for dealing with the successful containment until the fifth wave hit the city. While the net-zero policy maintained lower number of cases following rapid response, which may include extensive testing and contact tracing, local lockdowns, and travel restrictions, it also created a false assurance for the senior citizens to not get vaccinated over the span that resulted in one of the lowest inoculation rates amongst this section while the counter mega cities like Singapore and other economies had steadily progressed with vaccinating its older adults to prevent what is currently, in the fifth wave is haunting the Hong Kong older population with highest fatality rate in the world at the moment. Previously, while the city was well-insulated from the virus, identifying a single case in a care home required transferring the infected to be admitted in the hospital and all other residents in close contact to quarantine facilities and isolating them for at least 14 days to avoid adding to the number of cases through cross-contamination. The Department of Health overlooked the necessity to urge the older people to be vaccinated during a reasonable timeframe or create alternate emergency disaster prevention planning in case of widespread situation like the ongoing wave. The vaccination rate for 65 and older was looming just over 40 percent while the percentage for the 80 years and above were <20 percent just before the fifth wave. However, in the present situation, with the cases spiraling, the older people are facing the greatest difficulties, as quarantine facilities have reached capacity and hospital beds have exceeded the 90% bed occupancy threshold.

Several older people, among the reported 12,000 people who were waiting to be admitted to the facilities, were left on the streets in the open due to a lack of hospital beds and were not permitted to enter care homes either for fear of disease transmission on February 18 (6). This inhuman situation is absolutely inexcusable. On February 19, the government announced that arrangements have been made to incorporate newly constructed public housing estates, hostels, and sports centers into quarantine spaces capable of housing up to 20,000 additional COVID patients, but these will also become largely redundant if the current rapid transmission rates are not controlled (14).

Since the zero COVID policy works best when the number of infected cases is limited, as has been demonstrated in Hong Kong over the last 2 years, the current scenario with a high number of infected cases is having a direct impact on the older population, who are the most at risk of contracting the virus. At the moment, with over 50,000 cases reported daily in the first 3 days of March, authorities are pushed to reconfigure their isolation strategies due to the large influx of positive cases and shortage of quarantine spaces. This clearly highlights that more focus is needed toward the better facilitation of the existing living arrangements than staging temporary solutions which can never contain such immense numbers during any given outbreaks of this scale. So far, the pandemic's fifth wave has hit 755 elderly homes. COVID-19 infections have been confirmed in 72 percent of the city's elderly homes, affecting 9,800 people, or 13 percent of its total residents. Staff personnel have also been infected at a rate of 9.5 percent. At least 680 people died as a result of their infections. To get a sense of the severity on a single day, Larry Lee Lap-yee, the Hospital Authority's chief manager for integrated clinical services, reported 136 deaths on March 4th (15). There were 76 men and 60 women among the deceased, ranging in age from 29 to 102. There were 131 senior individuals aged 65 and up among them (96 percent of the total fatalities), with 73 living in elderly homes (53 percent of the total fatalities). Only 17 of them received two doses of the COVID-19 vaccination, while the remaining 21 received only one dose. The rest of the group were not vaccinated.

According to the Center for Health Protection, more than 90% of those who died in the fifth wave did not receive two doses of vaccines. While the lowest inoculation rates among the older people in Hong Kong contributed largely for such high fatality rates. However, it is critical to consider how the city's highly dense structure aided in the rapid transmission, as evidenced by the rapid spread from 40 reported COVID-19 infected care homes on February 20 (6, 7) to a whopping 755 care homes registered in a 2-week interval on March 4 (15). With the primary objective of containing the outbreak with the least possible damage, Hong Kong's policy has shifted from “net-zero” to “dynamic-zero,” to currently focusing on reducing the number of infected cases and admitting only cases exhibiting serious symptoms to public hospitals to support the overburdened medical service systems.

According to the recent press release on March 09 (16), the government will add community isolation and respite facilities as part of its efforts to increase support for older people afflicted with COVID-19. All elderly care home patients must now be inoculated (at least the first dose) by March 18th, according to the recent mandate. The authorities will take the lead in implementing closed-loop management in residential care homes that have not yet had an infection case, as well as providing dedicated hotels and vehicles for staff caring for the elderly, in order to prevent them from contracting the novel coronavirus after returning home or entering the community.

## Conclusion

The pandemic situation, which had been under control and almost dormant in Hong Kong for the past 2 years, is now wreaking havoc and putting a tremendous strain on the healthcare system. The variant Omicron, which predominates in the fifth wave in Hong Kong, has adversely impacted children and the older population (9). As observed in Hong Kong's poorest district, Sham Shui Po, it was one of the first districts to be affected by COVID-19 since the first outbreak (7, 17) and quickly became the hotspot with one of the highest number of cases in the city due to its ultra-dense morphology, which included the presence of mostly poor migrants and low-income workers (18, 19) living in sub-divided flats with poor hygiene, amongst other factors. The older population in Hong Kong, which is already vulnerable, is at an even greater risk because they are disproportionately vaccinated against the virus, which could otherwise serve as a shield. This makes elderly care homes a potential disaster zones in the city, due to the poorly designed indoor environments with inadequate ventilation, cramped spaces, and design that is not resistant to such outbreaks, which house several older people who generally lack the physical strength to fight back against the disease.

This is an essential reminder, in my opinion, that these facilities need to be updated with new design frameworks that considers every aspect, including lessons learned from the ongoing pandemic, and help to turn them into positive rather than unfavorable spaces. Fundamental reforms to health care systems, antiquated regulations, and the built environment are necessary and must be implemented. The current situation quite strongly highlights the shortcomings of the care homes in effectively dealing with the outbreak, as exposed in the fifth wave with no possibilities to accommodate additional cases for isolation and avoid cross contamination at the same time in the care homes and it is imperative that we learn from this and take necessary actions to better prepare for similar disasters in the future. It is a global challenge and allocating resources to future-proof our cities is critical, as the number of pandemics, epidemics, and natural disasters is likely to increase in the coming years as a result of human interference and climate change. A more “place-based” or territorially responsive strategy to pandemic preparedness is required at this time, given the challenges the government is facing in maintaining the zero-COVID policy. Further in-depth follow-up analysis can be carried out to provide more evidence to validate the outlines that have been emphasized.

In a densely urbanized metropolis like Hong Kong, the interrelationship between the built environment, urban population density, and overall public health outcomes during emergencies such as the COVID-19 pandemic offers significant opportunities for research and development. It will require an interdisciplinary approach to developing evidence-based new models of disaster management and policies, involving expert opinions from policymakers, urban planners, health practitioners, social designers, and architects, among others, because the challenges are wicked and highly entangled.

## Author Contributions

The author confirms being the sole contributor of this work and has approved it for publication.

## Conflict of Interest

The author declares that the research was conducted in the absence of any commercial or financial relationships that could be construed as a potential conflict of interest.

## Publisher's Note

All claims expressed in this article are solely those of the authors and do not necessarily represent those of their affiliated organizations, or those of the publisher, the editors and the reviewers. Any product that may be evaluated in this article, or claim that may be made by its manufacturer, is not guaranteed or endorsed by the publisher.
